# Identification and Distinction of Tenocytes and Tendon-Derived Stem Cells

**DOI:** 10.3389/fcell.2021.629515

**Published:** 2021-04-16

**Authors:** Yuange Li, Tianyi Wu, Shen Liu

**Affiliations:** Department of Orthopaedics, Shanghai Sixth People’s Hospital, School of Medicine, Shanghai Jiao Tong University, Shanghai, China

**Keywords:** tenocytes, TDSCs, identification, biomarkers, tenogenesis

## Abstract

Restoring the normal structure and function of injured tendons is one of the biggest challenges in orthopedics and sports medicine department. The discovery of tendon-derived stem cells (TDSCs) provides a novel perspective to treat tendon injuries, which is expected to be an ideal seed cell to promote tendon repair and regeneration. Because of the lack of specific markers, the identification of tenocytes and TDSCs has not been conclusive in the *in vitro* study of tendons. In addition, the morphology of tendon derived cells is similar, and the comparison and identification of tenocytes and TDSCs are insufficient, which causes some obstacles to the *in vitro* study of tendon. In this review, the characteristics of tenocytes and TDSCs are summarized and compared based on some existing research results (mainly in terms of biomarkers), and a potential marker selection for identification is suggested. It is of profound significance to further explore the mechanism of biomarkers *in vivo* and to find more specific markers.

## Introduction

Tendons, the connective tissue that connects muscle to bone, improve stability and promote movement, but are also particularly vulnerable to damage (Elliott, 1965). Tendons are generally hypocellular, thus may lack of adequate cellularity for sufficient healing (Tang et al., 2016). The healing of tendon injuries is very slow and scar tissue forms easily, which reduces the toughness of the tendon, thus affecting the mechanical properties of the tendon (Evans, 2012). It is more likely for injured tendons to be damaged again after repair (Longo et al., 2011). In addition, chronic injury can result in many degenerative changes of the tendon, such as single or simultaneous lipid deposition, proteoglycan accumulation and tissue calcification, and finally leads to tendinopathy (a chronic tendon injury) (Kannus and Józsa, 1991). Moreover, tendon adhesion is a common complication after tendon injury, which can seriously affect the patient’s limb function. Our understanding of the mechanism of tendon adhesion is still limited (Wong and Peck, 2014), making tendon adhesion a complex clinical problem.

Bi et al. (2007) reported to isolate a cell population termed tendon stem/progenitor cells (TSPCs) (also regarded as tendon-derived stem cells (TDSCs)] from human and mouse in 2007, which shows characteristics of stem cells such as multipotency, clonogenicity, and self-renewal capacity. Since then, how to effectively activate the endogenous or transplanting TDSCs and promote the proper differentiation has become a new way to treat tendon regeneration after injury (Leong and Sun, 2016). Although there are many studies on TDSCs, the identification of TDSCs is lack of specificity and consensus (Walia and Huang, 2019). Besides, the isolation of tenocytes and TDSCs follows the similar cell extraction protocol. However, in most of the literature, no distinction is made between the two types of cells (Cao et al., 2002; Wang et al., 2008; Chen et al., 2012). Some authors suggest the acquired cells are tenocytes while others think of them as TDSCs, making a conflict about what the cells really are. And there is a lack of comparison of tendon derived cells, with only three studies directly comparing tenocytes and TDSCs in the horse (Williamson et al., 2015),the rabbit (Zhang and Wang, 2010), and the mouse (Lee et al., 2018). This leads to a lack of accurate awareness between tenocytes and TDSCs, which is not conducive to further research.

This article aims to discuss the marker expression, morphology and application of tenocytes and TDSCs based on current documents, and summarize the potential methods for distinction and identification of tenocytes and TDSCs. According to the limitation of existing research, the future direction is proposed.

## Tenocytes

Tenocytes are tendon-specific fibroblasts and are considered to be made up approximately 95% of tendon tissue (Kannus, 2000). How tenocytes are produced during embryo development remains unclear (Ma et al., 2018). Tenocytes are transformed from tenoblasts (Kannus, 2000). The tenoblasts are round cells with large, oval nuclei (Luesma et al., 2020). Mature tenocytes are spindle-shaped, 80–300 in diameter, and the processes are long and thin (Franchi et al., 2007). The cellular processes extend away from the body of the cell, making the tenocytes look like spiders from transection view. Tenocytes are laid between collagen fibrils and are in charge of the production of extracellular matrix (ECM) as well as maintenance and restore of tendon tissue (Hess et al., 1989). Tenogenic differentiation markers are commonly used for the identification of tenocytes.

Scleraxis (Scx) and Tenomodulin (TNMD) are confirmed to be relatively specific molecular markers of tendons and make a central role in the development and maturation of tendons (Aslan et al., 2008). Scx is a basic Helix-Loop-Helix (bHLH) transcription factor, found by Cserjesi et al. (1995) using the yeast two-hybrid system for cell-type-specific proteins in 1995. Brown et al. (1999) used whole-mount *in situ* hybridization to show that Scx could be detected in the blastocyst at 3.5 day at the earliest and expressed in the mesoderm at 7.5 day, while after 11.5 day, Scx was confined to the cartilaginous primordia of axial and limb bones and the primordia of connective tissues such as tendon and ligament. Scx is widely expressed in connective tissue cells and is a marker of connective tissue (Muir et al., 2005; Levay et al., 2008). Murchison et al. (2007) produced Scx^–/–^ mice using gene knockout techniques, which showed severe defects in force-transmitting tendon of paws, backs and tails. Lessened and disorganized tendon matrix and disorder at the cellular level were also found with histological observation. Scott et al. (2011) constructed a murine patellar tendon injury model, and results of qPCR revealed that the expressions of Scx and TNMD were significantly up-regulated, while the latter lagged behind the former in time, indicating that Scx had effect on TNMD. Thus it can be seen that Scx begins to be expressed at the early stage of embryonic development and produces a regulatory effect on the process of tendon maturation.

Tenomodulin is a kind of type II transmembrane protein first reported by Shukunami et al. (2001). TNMD and Chondromodulin-I (ChM-I) are similar in structural configuration, sharing a homologous domain C-terminus (Hiraki et al., 1997). Its name comes from tenocytes, because TNMD is mainly expressed in tendons, as well as in ligaments, epimysium and skeletal muscle (Brandau et al., 2001). As mentioned above, TNMD is identified as a late tendon differentiation marker compared to Scx for early tendon differentiation (Takimoto et al., 2012). Oshima et al. (2004) used the adenovirus expression system to force expression and the following secretion of 116 amino acids at the TNMD C-terminus in human umbilical vein endothelial cells (HUVECs) and evaluate the anti-angiogenetic properties of TNMD. The result showed that the angiogenetic ability of HUVECs was dramatically impaired. Meanwhile, *in vitro* studies have found that TNMD and ChM-I can significantly reduce the vascular density in human melanoma. Therefore, it is speculated that TNMD functions in inhibiting angiogenesis in the highly expressed non-vascular tissues such as ligaments and tendons. Docheva et al. (2005) bred TNMD-defect mice by gene knockout technology, and found that the proliferation of tenocytes in mice was observably reduced and cell density was decreased. ECM, including collagen type I, II, III, VI, and all sorts of proteoglycan content is not affected, but the difference between collagen fiber diameter increased, appearing fibers with an obviously large diameter. It can be seen that TNMD can regulate the proliferation of tenocytes and the maturation of collagen fibers.

Besides Scx, Mohawk (Mkx) is another important regulator of TNMD, since a markedly decreased expression of TNMD could be observed in Mkx^(–/–)^ mutant mice (Liu et al., 2010). Mkx, identified by Anderson et al. (2006), is a homobox gene that is most closely related to IRX gene. The absence of Mkx can lead to heterotopic ossification of the Achilles tendon within a month after birth in mice, and the ossification gets worse with age (Liu et al., 2019). Moreover, Mkx^(–/–)^ mutant mice showed abnormal tendon sheath and the defect of tendon collagen fibers, revealing that Mkx is an important regulator of tendon development (Liu et al., 2010).

Type I collagen (Col I) and type III collagen (Col III) are other two matrix genes for tenogenic differentiation. Col I is originally synthesized as procollagen, a precursor form, consisting of two identical pro-α1(I) and one pro-α2(I) chains, encoded by COL1A1 and COL1A2 gene, respectively (Lu et al., 2019). Col III is made up of three identical pro-α1(III) encoded by COL3A1 (Gelse et al., 2003). Tenocytes compound mainly Col I and Col III (Hong et al., 1979) and the major problem encountered during long-term tenocytes culture is the decrease in the synthesis of Col I and Col III (Birk and Trelstad, 1986). Güngörmüş and Kolankaya (2008) used immunofluorescence staining technique to reveal that Col I mainly existed around the nucleus and Col III was more dispersed in the cytoplasm. Col I and Col III play a strong part in development and healing of tendons, however, an excessive amount of collagen will lead to tendon adhesions (Wong et al., 2009). Tendons are mainly composed of Col I and the fibers are arranged along the tendon axis (Franchi et al., 2007). Col I and Col III are co-distributed in the tendon bundle and the connective tissue at 14 days of development in the immature tendon (Birk and Mayne, 1997). During the fibroblastic phase (days 3–14) of tendon healing, the initial deposition of Col III occurs in a disorganized manner and then reassembles into a longitudinal structure (Wong et al., 2009). Col III is subsequently replaced by Col I in the remodeling phase (beyond day 10) (Myer and Fowler, 2016). Sakabe et al. (2018) found that ECM deposition reduced and disordered Col III failed to convert to ordered Col I at the tendon injury site of Scx^–/–^ mice, resulting in weak tendon healing strength and an increased degree of tendon adhesion. Riederer-Henderson et al. (1983) identified the type of collagen synthesized by chicken embryo tendon cells and fibroblasts of the synovium of sheathed tendons by isotope labeling *in vitro* and immunohistochemistry. It was concluded that tendon cells only synthesize Col I, while fibroblasts synthesize Col I and Col III.

Thrombospondin-4 (TSP-4 or Thbs4) and cartilage oligomeric matrix protein (COMP) are members of thrombospondin gene family (Lawler et al., 1993). Hauser et al. (1995) found that pentameric TSP-4 consisted in bovine tendon ECM in 1995. It was shown by Södersten et al. (2006) that both TSP-4 and COMP existed in equine tendon and were unable to be parted under non-reducing conditions, indicating hetero-oligomers formed via connection of disulfide bridges. Frolova et al. (2014) found that larger tendon collagen fibrils were over-represented in Thbs4^–/–^ than wild-type mice. Thbs4^–/–^ mice had smaller soleus muscles and reduced grip strength of their hind and forelimbs compared with wild-type mice. It suggests that the deficiency of TSP-4 changed the regulation of the composition of the ECM in tendons and muscles. The function of COMP is unclear, but it is considered to provide integrity to the structure of ECM (Södersten et al., 2005). In addition, COMP and TSP-4 can be expressed as an answer to mechanical load (Södersten et al., 2007).

Decorin (DCN) is a member of small leucine-rich proteoglycans (SLRP) family, distributed in the connective tissues. Samiric et al. (2004) found that DCN was the core protein of about 80% proteoglycan in fresh tendons through amino acid sequence analysis. Svensson et al. (1995) found that DCN mainly interacted with Col I fibrils through 4 to 5 leucine-rich repeats to influence the diameter of fibrils and form thinner fibers. Elliott et al. (2003) studied the tail tendon of DCN knockout mice and found that the viscoelastic properties showed greater and faster stress relaxation in DCN^–/–^ mice compared to the control group. DCN also antagonizes the synthesis of pathological collagen and plays the neutralization by down-regulating TGF-β1 (Abbah et al., 2016).

Tenascin C (TnC) is an ECM glycoprotein existing in mature and growing tendons (Kardon, 1998). TnC binds to a variety of surface receptors, for instance, integrins and other components of ECM, and thus participates in the regulation of cell-matrix interactions (Jones and Jones, 2000). It suggests that TnC is instrumental in the correct localization and alignment of collagen fibers in the tendon (Mackie and Ramsey, 1996).

The tenogenic differentiation markers mentioned above are commonly used in previous studies for tenocytes. The markers selected for the identification of tenocytes in several different studies are listed in Table 1. However, the expression of these genes is not limited to tendons, they also expressed in other musculoskeletal tissues, including cartilage, fat, muscle and bone (Jo et al., 2019). Therefore, the specific markers of tenocytes remain unfound, which brings difficulties to find a concise method to identify tenocytes.

**TABLE 1:** Expression of positive and negative markers in tenocytes in different species.

**References**		**Scx**	**TNMD**	**Mkx**	**Col I**	**Col III**	**Col IV**	**TnC**	**DCN**	**a- SMA**	**vimentin**	**GDF- 8**	**FAP**	**FSP1**	**Sox9**	**Runx 2**	**Cell source**	**Species**
Wagenhäuser et al., 2012		+	−		+	+		+	+								Biceps tendons	Human
Kendal et al., 2020	Tenocyte A	+		+	+	+											Multiple tendons	Human
	Tenocyte B	+		+	+	+												
	Tenocyte C				+	+		+										
	Tenocyte D				+	+			+					+				
	Tenocyte E				+	+			+					+				
Xu et al., 2018		+			+	+		+		+	+		+	+			Achilles tendons	Mouse
Chen et al., 2012		+	+		+	+	+					+					Digitorum profundus flexor tendons and Achilles tendons	Mouse
Fleischhacker et al., 2020		+	+		+	+									+	+	Achilles tendons	Mouse
Vermeulen et al., 2019		+	+	+	+	+		+							+	+	Achilles tendons	Rat
Evrova et al., 2020			+	+	+	+		+	+	+							Achilles tendons	Rabbit
Riederer-Henderson et al., 1983					+	−											Flexor tendons	Chicken

## TDSCs

As stem cells, TDSCs are self-renewing and can differentiate into tenocytes and other types of cells (osteocytes, chondrocytes, and adipocytes) under different conditions. In terms of cell morphology, TDSCs isolated from different tissue and species sources are different. Some are spindle-shaped with an appearance of fibroblasts, while others are like enlarged triangles (Lui and Chan, 2011). The ECM of TDSCs is mainly composed of fibromodulin, biglycan, and other components, and the microenvironment constituted by these is significant for the proliferation and differentiation of TDSC (Rui et al., 2011). However, the niche (both regulators and anatomical locations), characteristics and the role of TDSCs *in vivo* are still unclear (Lui, 2013). Although TDSCs have been isolated from tendons and cultured *in vitro*, the origin is still a controversial topic, and no specific markers are found.

Mesenchymal stromal cells (MSCs) must express CD105, CD73, and CD90, and lack the expression of CD45, CD34, CD14, or CD11b, CD79alpha or CD19 and HLA-DR surface molecules (Dominici et al., 2006). It is one of the minimal criteria proposed by the Mesenchymal and Tissue Stem Cell Committee of the International Society for Cellular Therapy. Therefore, TDSCs meet the above labeling requirements (Lui, 2015). However, TDSCs and other MSCs and certain differentiated cells cannot be accurately distinguished by these markers (Alt et al., 2011). Ruzzini et al. (2014) isolated tendon-derived CD44+ cells, which were positive for the stem cell marker CD146 and STRO1, and reported them to be TDSCs. The exact function of most CD markers in stem cells is unclear, causing difficulties in identifying specific markers of TDSCs. A better comprehension of the *in vivo* function of MSC markers in TDSCs will help to select appropriate markers to distinguish TDSCs from tenocytes.

In addition to this, TDSCs are reported to express pluripotency markers, including Nanog, Oct-4, Sox2, nucleostemin, and SSEA-4 (Zhang and Wang, 2010; Tan et al., 2013). Nanog was reported and officially named by Chambers et al. (2003). Nanog belongs to the NK-2 gene of the ANTP superfamily, which is a transcription factor expressed by inner cell mass, primitive germ cells and embryonic stem cells (ESCs). Studies have shown that Nanog not only help maintain the undifferentiated state of ESCs, but also promote cell proliferation (Hyslop et al., 2005). Oct4 (Octamer-binding transcription factor) is named for its ability to bind to DNA of octamer modes. Currently, the prevailing view is that Oct4 plays a nodal role in the transcription factors of somatic reprogramming, and no other core or auxiliary transcription factors can replace the function of Oct4 (Radzisheuskaya and Silva, 2014). Sex-determining region Y-box 2 (SOX2) identifies and combines the promoter of various target genes to transactivate or inhibit their expression through transactivation domain to regulate various physiological processes (Nowling et al., 2000). SOX2 makes a critical difference in maintaining the stem cell phenotype of ESCs during embryogenesis. What should be of concern is that SOX2, as one of the Yamanaka factors, together with the ectopic expressions of Oct4, Klf4, and C-MYC, transforms mouse embryonic fibroblasts into induced pluripotent stem cells (iPSCs) (Takahashi and Yamanaka, 2006). Nucleostemin was initially identified as a gene enriched in human cancer cells and neural stem cells (NSCs) (Tsai and McKay, 2002), and was later found to be highly expressed in several kinds of other stem cells (Baddoo et al., 2003). Nucleostemin, located in the cellular nucleolus, is a guanosine triphosphate binding protein, which can sustain the proliferation of stem cells and inhibit their differentiation to mature cells, and is mainly involved in maintaining the proliferation, differentiation, cell cycle process and renewal of stem cells and tumor cells (Tsai, 2014). Stage-specific embryonic antigen 4 (SSEA4), a core structure of globulin series carbohydrates, has been reported to be a cell surface marker to define a variety of human pluripotent stem cells, including ESCs (Rao et al., 2007) and MSCs (Wakao et al., 2012). However, the role of SSEA4 in the establishment and maintenance of ESCs and iPSCs is unclear (Hamamura et al., 2020). At present, no study has directly described the specific expression of these ESC markers in TDSCs. There is also a lack of information on the relational role of these ESC markers in the pluripotency and self-renewal. Nucleostemin, OCT-4, Nanog, and Sox2, as nuclear proteins, limit their application to be prospective isolation markers in clinical trials or practices. Surface marker SSEA4, may be more useful if proven to be effective.

In different studies, the marker expression of TDSCs are discrepant, which are affected by the cell source, cell isolation and separation procedures (Lui, 2015). Positive or negative expressions of TDSCs markers in different species are listed in Table 2.

**TABLE 2:** Expression of positive and negative markers in TDSCs in different species.

**References**	**Pluripotent markers**								**CD markers**														**Tenogenic markers**									**Cell source**	**Species**
	**nucleo stemin**	**Nanog**	**Oct- 4**	**Sox2**	**SSEA- 1**	**SSEA- 4**	**c- Myc**	**Sca- 1**	**CD 18**	**CD 31**	**CD 34**	**CD 44**	**CD 45**	**CD 73**	**CD 90**	**CD 90.2**	**CD 105**	**CD 133**	**CD 146**	**CD 166**	**Stro- 1**	**Nestin**	**Col I**	**Col II**	**Col III**	**SCX**	**TNMD**	**TnC**	**Comp**	**Aggrecan**	**αSMA**		
Tsai et al., 2013					−						−	+	−	+	+		+	−		+												Shoulder rotator cuff	Human
Kohler et al., 2013														+	+		+		+		+		+		+		+	+				Achilles tendons	Human
Yin et al., 2010									−		−	+			+		+		+													Achilles tendons	Human
Zhang and Wang, 2013a	+	+	+			+																	+					+				Patellar tendons	Human
Lee et al., 2012											−	+	−	+	+		+		−		−											Patellar tendons	Human
Rui et al., 2010										−	−	+			+								P0 P3−	−			+	+		+	+	Flexor tendons	Rat
Liu et al., 2013	+		+			+																										Flexor tendons	Rat
Chen et al., 2014												+			+																	Achilles tendons	Rat
Tan et al., 2013	+	+	+	+				+				+							+													Patellar tendons	Rat
Holladay et al., 2016										+	−	+			+								+	+		+	+					Patellar tendons	Rat
Alberton et al., 2015		+						+				+		+		+	+		+			+					+					Tail tendons	Mouse
Bi et al., 2007								+			−	+	−			+							+			+			+			Patellar tendons	Mouse
Mienaltowski et al., 2013								+	−		−	+			+			−														Achilles tendons	Mouse
Zhang and Wang, 2013b	+		+		+																											Patellar and Achilles tendons	Mouse
Zhang and Wang, 2010	+		+			+																										Patellar and Achilles tendons	Rabbit
Tao et al., 2015	+		+			+																										Patellar tendons	Rabbit
Yang et al., 2013	+	+	+																													Patellar tendons	Rabbit
Yang et al., 2018															+		+						+		+						+	Achilles tendons	Porcine
Lovati et al., 2011			+			+	+																									Superficial digital flexor tendons	Horse
Yang et al., 2016												+											+	−	+			+				Achilles tendons	Fetal bovine

Yin et al. (2016) found a subpopulation of nestin^+^ TS by single-cell analysis in 2016. Nestin, a type of intermediate filament protein specifically expressed in neuroepithelial stem cells (Wiese et al., 2004),was found to be of significance to self-renewal stem cells (Park et al., 2010). The study demonstrated that the expression of nestin was related to tendon differentiation and nestin^+^ cells were involved in endogenous tendon injury repair. Nestin^+^ cells showed stronger self-renewal, tenogenesis and colony formation abilities than cells treated by nestin knockdown. The authors further verified that knockdown of nestin did harm to tendon healing and regeneration in a patellar tendon defect model *in vivo*. The cells arranged randomly and the collagen fibril was misaligned. In summary, it is indicated that nestin is a characteristic marker of TSCPs and new insights are provided into the study of TSCPs.

Harvey et al. (2019) made great progress in the research of cellular and molecular mechanisms of tendon regeneration and suggested that the Tppp3+/pdgfra+ subpopulation possibly indicated TDSC status. Tubulin polymerization-promoting protein family member 3 (Tppp3), is reported to be a specific marker of the differentiating synovial joints and tendon sheath (Staverosky et al., 2009). Harvey et al. (2019) used single-cell RNA sequencing to classify all the cells in the patella tendon of adult mice and obtained eight clusters. One of them, an unknown cluster was identified as TDSC, which was enriched for Tppp3+. The authors used lineage tracing to prove that Tppp3+ cells were activated after tendon injury and were involved into tendon healing. Transcriptomic analysis revealed the enrichment of PDGFR in the putative TDSCs. PDGFRa, platelet-derived growth factor receptor alpha, and other PDGF signaling components are confirmed to promote the proliferation of TDSC and facilitates tendon fibrosis and regeneration.

These are two studies that had identified a potential marker of TDSCs and explored their effects *in vivo*. Although there is still some left to further elucidate the complex role of the microenvironment, the latest studies have made a significant leap in the research of TDSCs and tendon regeneration (Titan and Longaker, 2019).

## Comparison of Characteristics of Tenocytes and TDSCs

### Subpopulations

Kendal et al. (2020) performed scRNA-seq and CITE-seq integrated analysis of cells from tendon samples, and eight distinct transcriptomic clusters were found, including five tenocyte clusters (initially labeled “Tenocyte A–E”), monocytes, lymphocytes and a group of combined endothelial cells. Part of phenotypes of Tenocyte A–E is shown in Table 1. The authors failed to detect a stem cell population in human cells. Although the up-regulation of PDGFRA, TPPP3, and other genes which are expressed in TDSCs were displayed in Tenocyte D and Tenocyte E clusters, the article raised some questions about the presence or absence of TDSCs.

It has been reported that mouse TDSCs contained two subpopulations, CD105-positive cells and CD105-negative cells (Asai et al., 2014). Compared with CD105-positive cells, CD105-negative cells showed greater cartilage potential *in vitro* and induced greater cartilaginous degeneration in mice.

### Factors Influencing Characteristics

#### *In vivo* and *in vitro*

As mentioned above, many achievements have been made in the expression of tenogenic differentiation markers of tenocytes *in vivo* and *in vivo* and their roles *in vivo*. However, *in vivo* studies of MSC markers and ESC markers in TDSCs are lacking. Immunohistochemical staining showed the absence of ESC markers (nucleostemin, Nanog, Oct-4, and SOX2) in all intact rat patella tendon cells (Tan et al., 2013). The existing *in vitro* markers are not effective *in vivo*, suggesting the need for more research on TDSCs.

#### Cell Passaging

Phenotypic drift of the cells leads to changes in common cell markers over multiple passages. After two passages, the expression levels of Col I and Col III in human tenocytes were significantly decreased (Mazzocca et al., 2012). DCN, Scx, and TnC gradually declined over six passages. In addition, with progressive passage, tenocytes became more rounded. Cells of the sixth passages did not have the fibroblast characteristics any more showed in the previous two passages. The cellular processes were longer and thicker of early passage compared with the sixth passage.

Similarly, the expression of Scx and TNMD decreased during passage in TDSCs (Tan et al., 2012). Meanwhile, the ability of chondrogenic, adipogenic, and tendinogenic differentiation of TDSCs decreased, and the ability to differentiate into osteogenic lineage increased. Moreover, the surface expression of CD73 and CD90 in TDSCs was down-regulated with passaging. It showed no difference with previous reports that MSCs began to lose their stem cell properties with passaging *in vitro* (Izadpanah et al., 2008) and markers distinguishing MSCs from fibroblasts were down-regulated with passaging (Halfon et al., 2011).

#### Culture Conditions

Phenotypes can be influenced by culture conditions. The expression of markers can be induced or inhibited by growth factors in fetal bovine serum, seeding densities, oxygen tension and mechanical loading. For instance, human TDSCs cultured in 5% oxygen had significantly greater cell proliferation and expressed higher levels of nucleostemin, Nanog, and SSEA-4 (Zhang and Wang, 2013a).

### Applications

The ability of TDSCs to self-renew and their potential to differentiate into tenocytes are expected to help reconstruct the function and structure of damaged tendons, and the application in the cure of tendon injuries has already been explored. TDSCs were first used to accelerate tendon repair in a rat patellar tendon window defect model in 2011 (Ni et al., 2012). The mechanical properties (elastic modulus and ultimate stress) of tendon treated with stem cell therapy were significantly higher than those without TDSCs. More research on TDSCs in tendon–tendon healing (Komatsu et al., 2016), tendon–bone healing (Shen et al., 2012; Lui et al., 2014b), and tendinopathy (Chen et al., 2014; Durgam et al., 2016) has followed. Biologically speaking, using autologous TDSCs to heal tendon injuries may be the most ideal choice. However, due to the limited number of TDSCs in normal tendon, it is hard to obtain a great quantity of TDSCs from self (Rui et al., 2010). It has been shown that allogeneic TDSCs can also facilitate tendon injury repair without inducing intense immune response and anti-inflammatory effects in mice (Lui et al., 2014a). However, the number of transplanted TDSCs decreased gradually over time, indicating that the role of allogeneic TDSCs was mainly through inducing cell differentiation rather than directly differentiating by themselves.

In contrast, the use of tenocytes in cell-based therapy has been much less reported. Wang A. et al. (2013); Wang et al. (2015) reported to utilize autologous tenocyte injection (ATI) as a treatment for severe, chronic resistant lateral epicondylitis and autologous tenocyte implantation for tendinopathy and partial-thickness rotator cuff tear in an elite athlete (Wang A.W. et al., 2013). Bucher et al. (2017) used ATI for the treatment of chronic recalcitrant gluteal tendinopathy. All of the treatment have had some effects.

The difference between the effects of the two types of cells in cell therapy is still unclear and needs further study.

### Direct Comparison

There is little literature that directly compares the two types of cells. The comprehensiveness of the comparisons in the literature varies and discrepancy exists between different species.

Zhang and Wang (2010) applied trypsin locally under microscope and collected detached cells using micropipette to separate TDSCs from tenocytes. They characterized the properties of rabbit tenocytes and TDSCs. Tenocytes tend to spread outward and are highly elongated, showing characteristic shapes of fibroblasts under confluent conditions. The cell colonies of TDSCs shape like pebbles, with smaller volume and larger nucleoli compared with tenocytes. It was shown that TDSCs proliferate faster than tenocytes. TDSCs expressed nucleostemin, Oct-4, and SSEA-4 while these markers were expressed lowly or barely in tenocytes. In addition, TDSCs had the ability of tri-lineage differentiation *in vivo* and *in vitro* that tenocytes essentially did not possess. It is a pity that the authors did not compare from more markers.

Williamson et al. (2015) first seeded the cells digested from equine tendon at different densities to isolate tenocytes (high density) and TDSCs (low density). The results of the tri-lineage differentiation assays suggested that this method might not be suitable for equine TDSCs and the authors then used differential adhesion of fibronectin to isolate TDSCs. However, the proliferation rates between the tenocyte and the putative TDSCs in 5% oxygen had no significant differences. In addition, they compared the expression of tenocytes and TDSCs by quantitative RT-PCR. Except for a higher expression of TSP-4 in tenocytes, they did not find other significant differences. Other markers included Nanog, Oct-4, CD90, CD73, CD34, CD144, TnC, Scx, Mkx, EGR1, and TNMD. If species specificity is excluded, the results suggested that this method may also not be an ideal way to separate TDSCs from tenocytes. Lee et al. (2018) made a comparison of characteristics of murine tenocytes and TDSCs. They used cloning cylinders and applied trypsin topically to isolate TDSCs. Tenocytes are large, tabular and fibroblastic, while TDSCs are smaller and rounder (Lee et al., 2018). Tenocytes formed large and sparse colonies, while the colony formation of TDSCs were denser and more compact. When compared with tenocytes, early tenogenic markers (Mkx and Scx) and stem cell markers (CD73 and Nanog) were expressed more highly in TDSCs, whereas TSP-4, TNMD, and TNC presented a lower expression in TDSCs than in tenocytes. Stem cell antigen-1 (Sca-1) and CD90 were expressed similarly in two cell types. The hematopoietic stem cell marker CD45 showed low expression in TDSCs, but evidently higher expression was observed in tenocytes. However, the authors merely made simple comparison and did not expound and further study the significance behind the results. They also analyzed the capability of tenocytes to differentiate into different cell lineages, and found that osteogenic differentiation and chondrogenic differentiation were observed in tenocytes, but tenocytes demonstrated no adipogenic differentiation. This conclusion contradicts the common belief that tenocytes are terminally differentiated cells (Viganò et al., 2017). We suspect that the characterization of the tenocytes was done with a mixture of tenocytes and TDSCs rather than “pure” tenocytes, which might have an effect on the presented data.

To sum up, although there are some different views, the mainstream opinion is as follows:

Markers commonly used for tenocytes are tenogenic differentiation markers, which are expressed in different degrees in both tenocytes and TDSCs. So the tenogenic markers are not indicators that directly differentiate between tenocytes and TDSCs. TDSCs have the potential of multi-differentiation and self-renewal, which are not available in tenocytes. Therefore, MSC and ESC markers and tri-lineage differentiation assays can be used to distinguish between tenocytes and TDSCs. A rough comparison of tenocytes and TDSCs is summarized in Table 3.

**TABLE 3:** Summary of general comparisons between tenocytes and TDSCs.

	**Tenocytes**	**TDSCs**
Morphology	Large, tabular and fibroblastic	Smaller and rounder
Markers	Mainly tenogenic markers	Mainly tenogenic markers, MSC markers and ESC markers
Multi-differentiation	−	+
Self-renewal	−	+
Applications	The difference is still unknown	

## Conclusion and Prospect

The research of tenocytes and TDSCs has been a hot topic in recent years. The isolation and identification of tenocytes and TDSCs have been a relatively neglected problem. In most literature, cells are extracted from tendons following a similar enzymatic digestion protocol and are monolayer-cell cultured in almost the same environment. The resulting cells theoretically contain both tenocytes and TDSCs. Some authors identify these tendon cells as tenocytes by tendon-specific markers, while others identify them to be TDSCs by stem cell markers. The distinction and identification between both types of cells is missing, which is appreciably rough and lacks of preciseness. Furthermore, the lack of specificity of these markers leads to no consensus on the identification of the two kinds of cells. Therefore, the markers selected in different research articles are not uniform and not fixed. For example, Rui et al. (2010) used flow cytometric analysis to test that the tendon-derived cells were positive for CD90 and CD44,and negative for the endothelial cell marker CD31 and hematopoietic stem cell marker CD34. These cells were considered as TDSCs. And for identification of porcine TDSCs, Yang et al. (2018) chose some other surface markers: CD105, CD90, Col I, Col III, and α-SMA. New advances of specific markers have been made, but have not been adopted widely in laboratory experiments. The discovery of nestin^+^ and Tppp3+/pdgfra+ population revealed the cellular and molecular role of TDSCs in tendon healing and regeneration, providing a new enlightenment of biomarker research.

The frequently selected markers in tenocytes and TDSCs can be seen in Tables 1, 2. Combined with the characteristics of the markers and the frequency of selection, for now, we recommend the use of Scx, TNMD, Col I for the identification of tenocytes. Scx and TNMD are early and late tenogenic markers of tenocytes, respectively. Col I is the principal constituent of tendons. And for the identification of TDSCs, we recommend to use CD90 [expressed in a subtype of fibroblasts (Ho et al., 2019)] and CD44 [specific surface markers of MSCs (Yang et al., 2016)] combined with the above markers. CD90 is a GPI-anchored cell surface protein and regulates fibrosis (Saalbach et al., 2000). CD44 is a complex transmembrane adhesion glycoprotein that is basically related to the key component of ECM and hyaluronic acid (Thorne et al., 2020). Since tenocytes and TDSCs are both capable of tenogenic ability, it may not be necessary for some research to separate the two types of cells. In some necessary cases, high-throughput technologies may be a better way to distinguish TDSCs from tenocytes, although this is limited by conditions. Therefore, it is urgent to further study the isolation method of tenocytes and TDSCs with wide adaptability.

Many results and cues about functions and expression of TDSCs are characterized and identified *in vitro*. However, the role of MSC and ESC markers in TDSCs *in vivo* is unclear. To better understand their *in vivo* function and expression, using overexpression and knockdown methods or transplanting in null animal models are crucial for better selection of markers associated with multipotency and self-renewal of TDSCs. The research on nestin and Tppp3/pdgfra provides a good model. At present, although there are few reports about the subpopulations of tenocytes and TDSCs, the expression of each marker may be co-expressed by different subpopulations of cells, which leads to the limitations of the current identification. Therefore, co-expressed and hierarchical markers will also be a research direction in the future.

There is still great need for specific markers of tendon cells and reliable markers which can label TDSCs *in vivo*. With the understanding of the expression, mechanism and influencing factors of markers *in vivo*, researchers can better choose markers with strong specificity. An accurate separation of tenocytes and TDSCs from tendon cells is also necessary to study the role of each cells in tendon healing, adhesions or degeneration. Unknown fields still remain to be explored in the study of the characteristics and markers expression of tenocytes and TDSCs, which is of great significance for the eventual application in the prevention and treatment of tendon related diseases.

## Author Contributions

All authors determined the subject. YL and SL performed the analysis. YL wrote the manuscript.

## Conflict of Interest

The authors declare that the research was conducted in the absence of any commercial or financial relationships that could be construed as a potential conflict of interest.
